# *Costus afer*: A Systematic Review of Evidence-Based Data in support of Its Medicinal Relevance

**DOI:** 10.1155/2019/3732687

**Published:** 2019-12-25

**Authors:** Daniel Boison, Cynthia Ayefoumi Adinortey, Godwin Kweku Babanyinah, Olga Quasie, Rosemary Agbeko, Gilbert Kofi Wiabo-Asabil, Michael Buenor Adinortey

**Affiliations:** ^1^Department of Biochemistry, School of Biological Sciences, University of Cape Coast, Cape Coast, Ghana; ^2^Department of Molecular Biology and Biotechnology, School of Biological Sciences, University of Cape Coast, Cape Coast, Ghana; ^3^Centre for Plant Medicine Research, Mampong-Akuapem, Ghana

## Abstract

*Costus afer* (*C. afer*) is a plant commonly known as ginger lily, spiral ginger, or bush cane. It is reportedly used in traditional medicine practice (TMP) to treat and manage many ailments including diabetes mellitus, stomach ache, arthritis, inflammation, and gout. These purported ethnomedicinal uses have triggered many research studies on the plant to amass scientific evidence. However, these research reports are scattered, and thus, this systematic review seeks to provide a comprehensive update on it covering its traditional uses, phytochemical and nutritional constituents, pharmacological activities, and toxicological effects. An online search was done using search engines such as Google Scholar, PubMed, and ScienceDirect from the period 1970 to 2019. The online search included the use of keywords, “*Costus afer* Ker-Gawl” or “*Costus afer*.” The search revealed that the stem and leaves of the plant contain substantial amounts of micronutrients and macronutrients. The leaves, stem, rhizomes, and roots of *C. afer* contain several steroidal sapogenins, aferosides, dioscin, and paryphyllin C and flavonoid glycoside kaempferol-3-O-*α*-L-rhamnopyranoside. Experimental studies on various parts of the plant showed bioactivities such as antihyperglycemic, hepatocellular protection, cardioprotection, nephroprotection, testicular protection, CNS depressant, analgesic, antiarthritis, antibacterial, and antioxidant. Based on these evident data, it is concluded that the plant could be used as an alternative and complementary therapy for many oxidative stress-related diseases, provided further scientific studies on the toxicological and pharmacological aspects are carried out.

## 1. Introduction

The use of herbal medicine to manage or cure diseases dates back to the Stone Age. There has been an advancement in pharmacological discoveries over the years that has resulted in the production of many synthetic drugs. This therefore reiterates the worth of ethnomedicinal plants for drug discovery. *Costus afer* Ker-Gawl (*C. afer*) with synonyms *Costus obliterans* Schum, *Costus anomocalyx* Schum, and *Costus insularis* Chev. is a rhizomatous herb commonly known as ginger lily or “bush cane” [1–4]. Almost every part of this plant is endowed with medicinal potential in diseases such as malaria, measles, diabetes mellitus, arthritis, and stomach disorders. In West Africa for instance, the succulent stem is chewed to quench thirst and also to treat cough and its accompanying sore throat [5, 6]. Various solvent extracts of the plant leaves, stem, rhizomes, and roots have been studied and reported to contain chemical compounds that could be useful in the alleviation of oxidative stress-related conditions [7–9].

A number of research studies have gone into finding bioactive compounds of plant origin with pharmacological properties to be used in the design of new drugs with lesser side effects. The several medicinal importance of *C. afer* makes it serve as one of the plants to attract this kind of research. However, information on its bioactivity, safety profile, and active principles is scattered. Implicitly, there is the need to collate these for easy accessibility that could enhance further research. This review therefore tends to provide a comprehensive update on this plant covering the botanical and ecological distribution, phytochemistry, pharmacological actions, and toxicological consequences associated with usage.

## 2. Search Method

**Figure 1:**
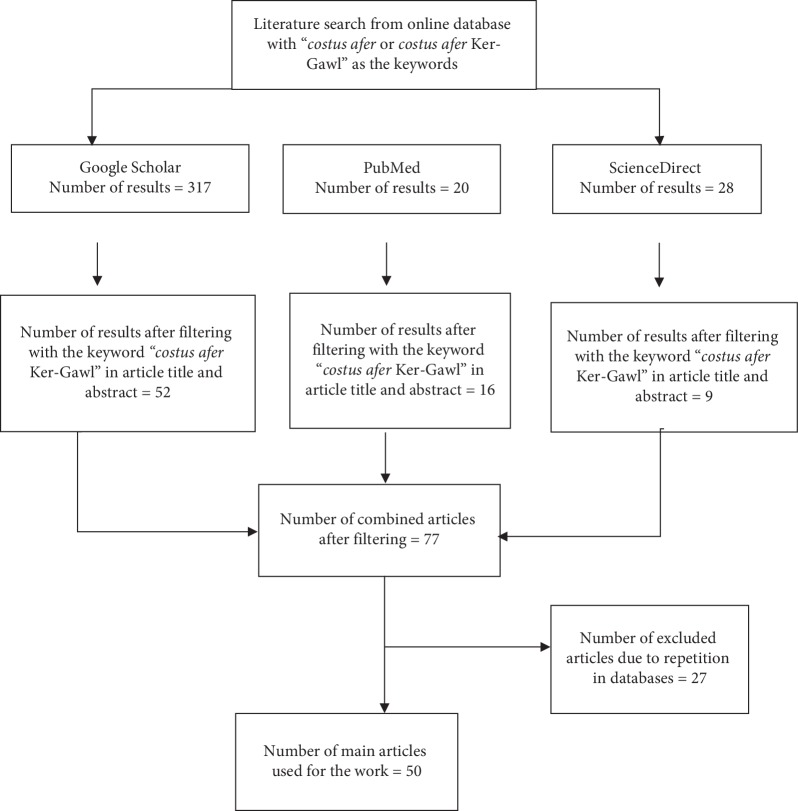
Flowchart of the systematic literature search scheme used.

An online literature search was performed in ScienceDirect, PubMed, and Google Scholar databases using keywords “*Costus afer* Ker-Gawl” or “*Costus afer*” with a year limit of articles published from January 1970 to October 2019 in accordance with the Preferred Reporting Items for Systematic Reviews and Meta-Analyses (PRISMA) guidelines. The search approach is illustrated in a flow diagram (Figure 1). The article selection was based on the following inclusion criteria: articles published in English and articles with keywords in the title, abstract, or full text and studies with isolated compounds from the plant. Articles that were retrieved were manually reviewed with the goal of identifying and excluding works that did not fit the inclusion criteria described. Articles with repeated data and duplication by the search engines were also excluded.

## 3. Results

### 3.1. Taxonomy, Ethnic Names, and Ethnopharmacological Importance

*Costus afer* Ker-Gawl belongs to the domain Eukaryota, kingdom Plantae, and the family Zingiberaceae now known as Costaceae [10]. It is of the genus *Costus* and species *afer* [11]. The various ethnic names to which the plant is referred to in certain dialects in West Africa are documented in Table 1. Traditionally, the plant has several medicinal uses, which are summarized in Table 2. The pharmacological significance that is attached to the use of the plant has led to numerous scientific research publications.

### 3.2. Botanical and Ecological Distribution

**Figure 2:**
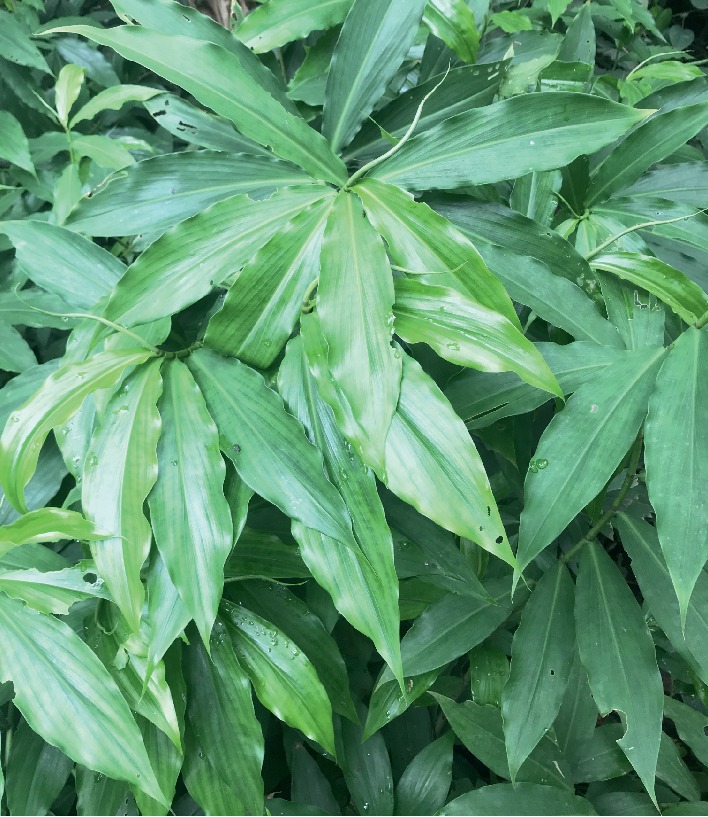
Leaves of *Costus afer*.

*Costus afer* Ker-Gawl is usually an unbranched tropical plant often seen as a herb with a creeping rhizome. It is a relatively small monocot shrub which is commonly found in humid and monstrous forests and riverside [11]. It is a perennial plant which can grow as tall as 4 m and bears white and yellow flowers [11, 24]. Its inflorescence is a highly compact, terminal, conical spike of about 2.5 cm to 7.5 cm long, sessile; bracts are oblong, convex, 3.5 cm long, densely imbricate, upper ones usually smaller; apex is truncate to rounded, green with purple markings, each subtending two flowers; bracteoles are boat-shaped, 2.5 cm × 1 cm; and keel is thick and ridged, pale green with pink markings and thin pink papery margin [5]. It has simple leaves, which are arranged spirally. The sheath is lobular, closed, and green with purple blotches. The ligule is about 4 to 8 mm long, which is leathery and glabrous. The leaf blade is elliptical to obovate of about 15 cm to 35 cm × 3.5 to 9.5 cm with culminate apex [18]. The margin is sparsely hairy with a bisexual and zygomorphic flower. A picture of *C. afer* leaves is provided in Figure 2.

*Costus* is pan tropical with about seventy species, of which forty are found in tropical America, twenty-five in West tropical Africa, and five in South-East Asia [25]. In Africa, the plant is found in the forest belt from Senegal to Ethiopia and in the East to Tanzania. In tropical West Africa, it is found in the rain forest and riverbanks of countries including Ghana, Sierra Leone, Senegal, Guinea, Togo, Cameroon, and Nigeria [5, 11].

### 3.3. Nutritional and Phytochemical Composition of *Costus afer*

**Figure 3:**
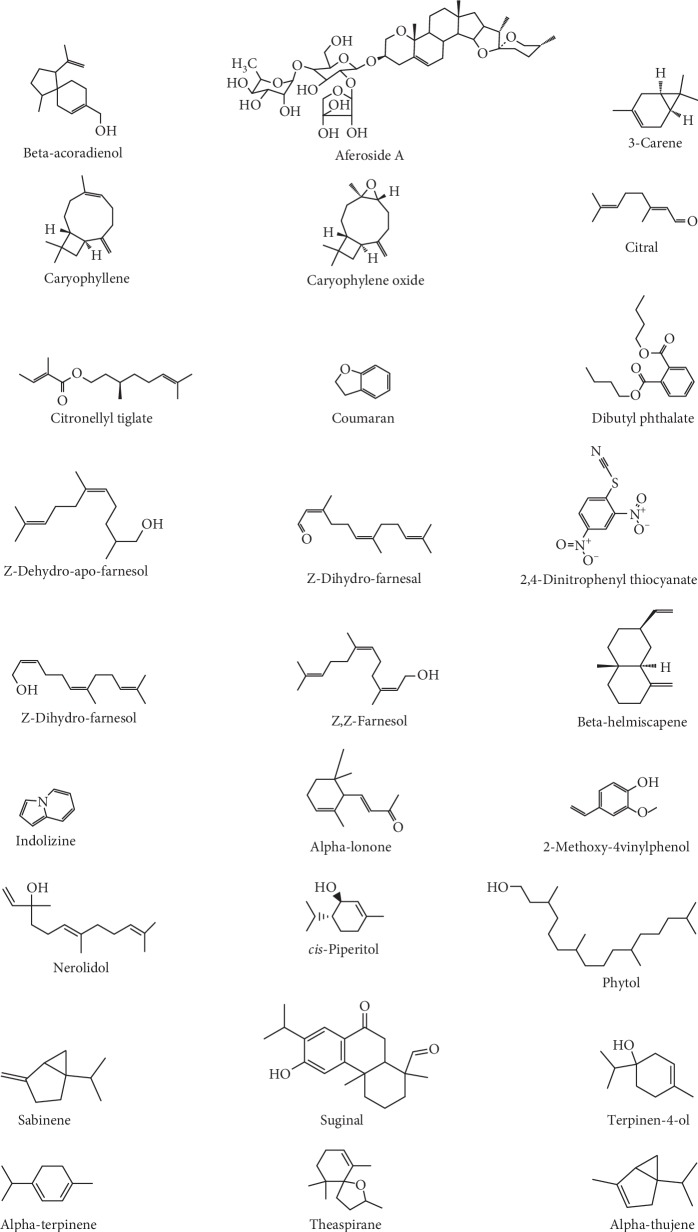
Chemical structures of some selected phytocompounds from different parts of *Costus afer*.

*C. afer* is used by the local folks, due to its nutritional and medicinal properties. This involves the use of the plant parts such as leaf, stem, and the rhizome in preparation of food [7, 18]. The proximate analysis of different parts of *C. afer* shows the presence of both macro- and micronutrients [26, 27]. Both the leaves and stem are rich in macronutrients such as carbohydrate, crude protein, fat, ash, moisture, and a good source of fiber. There are also reports of the presence of certain vital nutrients such as vitamins B (1, 2, 3, 6, and 12), E, and C in the leaves [28]. The oil extracted from the plant is made up of 78% saturated fatty acids and 22% unsaturated fatty acids [28]. The phytochemical analysis of the leaves, stem, and the rhizome of this plant in different solvents shows the presence of alkaloids, phenols, saponins, triterpenes, tannins, and glycosides [2, 29, 30]. These phytochemicals and nutrients may justify the nutraceutical use of the plant [4]. Research on the chemical identification and isolation of bioactive compounds from *C. afer* has been carried out, and this has led to the elucidation of structures from different parts of the plant [21, 31, 32]. For instance, the rhizome is reported to contain steroidal saponins such as diocin, paryphyllin C, aferoside B, and aferoside C. Kaempferol-3-O-R-L-rhamnopyranoside, which is a flavonoid glycoside, has also been identified from the aerial part of the plant [31]. Additional aferoside A [21] and aferosides B and C [32] have been isolated from the roots of *C. afer*. The structures of some compounds reported to be found in *C. afer* leaves, stem, rhizome, and roots are shown in Figure 3.

### 3.4. Pharmacological Activities of *Costus afer*

#### 3.4.1. Pancreatic Protection, Antidiabetic Property, and Hypolipidemic Effect

Diabetes mellitus is a chronic hormonal and metabolic disorder that is characterized by a persistent increase in blood glucose levels. In an alloxan-induced rat model, there was a significant reduction in blood glucose level when *C. afer* leaf extract at concentrations of 375, 750, and 1125 mg/kg and the control drug (glibenclamide (5 mg/kg)) were orally given [33]. A study by Ezejiofor and colleagues reported in 2014, 2015, and 2017 showed that *C. afer* leaf and stem extracts are able to reverse histopathological damage of pancreatic *β*-cells in alloxan-induced diabetes mellitus [34–36]. These reports are consistent with the work by ThankGod et al. [1], reporting on the evidence of regeneration of islet cell on the administration of *C. afer* stem extract to streptozotocin-induced rats. According to a report by Ezejiofor and colleagues published in 2015 [36], oral administration of 750 and 1125 mg/kg of *C. afer* leaf extract produced a more prominent regeneration and repopulation of islet cells. The same research group in 2017 in a histopathological study of *C. afer* stem extract on alloxan-induced damaged pancreatic cells noticed an organ protective effect [34]. This therefore indicates that *C. afer* leaf and stem extracts have pancreatic islet cell protective and regenerative effect that could be explored in managing type I diabetes mellitus.

Other studies have also attempted to elucidate mechanisms through which the *C. afer* leaf and stem extract may be exhibiting their antihyperglycemic effects. In an *in vitro* study involving different solvent extracts of *C. afer* leaf, stem, and rhizome on the activities of alpha-glucosidase and alpha-amylase enzymes, there was a significant inhibition of these enzymes. Ethyl acetate rhizome and methanol leaf extract exhibited the highest inhibitory effect with an IC_50_ of 0.10 and 5.99 mg/mL, respectively [37].

Glucose transport in 3T3-L1 adipocytes is a recognized *in vitro* model that represents a critical experiment for glucose utilization and disposal in mammals. In a study reported by Anaga and colleagues in 2004 [19], *C. afer* leaf extract displayed a high capacity to transport glucose at very low concentration (10 *μ*g/ml). When the effect of insulin at a concentration of 340 nM and extract at 10 *μ*g/ml concentration was compared, the extract caused about 96% glucose uptake compared to insulin in 3T3-L1 adipocytes. *C. afer* is also reported to significantly decrease plasma glucose after 30 to 60 minutes of oral administration than the reference drug tolbutamide.

It is clear that several studies attest to the fact that *C. afer* acts as an antidiabetic agent via biochemical mechanisms including restitution of pancreatic *β*-cell function, amelioration of insulin resistance by sensitizing receptors, inhibition of liver gluconeogenesis, enhanced glucose absorption, and inhibition of G-6-Pase, *α*-amylase, and *α*-glucosidase activities. The stem and roots are reported to contain several bioactive compounds [21, 32] with diosgenin and aferosides A, B, and C named as the most likely compounds responsible for the antidiabetic properties of *C. afer* [33]. Diosgenin ameliorates insulin resistance by increasing glucose usage and intracellular glycogen synthesis [30].

The concentration of lipids such as triacylglyceride (TAG), total cholesterol (TC), very low-density lipoprotein (VLDL), and low-density lipoprotein (LDL) is highly regulated to avoid certain clinical conditions such as steatosis. This condition occurs when there is abnormal retention of lipids within a cell as a result of impairment in the normal synthesis and degradation of fats. Accumulation of these fats is often associated with disorders and diseases such as diabetes mellitus, obesity, and hepatitis C. When the body is unable to control fat regulation, there is the need for extracellular regulation, which includes the use of a natural product such as *C. afer*. In both carbon tetrachloride-induced model [38] and streptozotocin-induced diabetic rat model [39], there was a significant rise in the TAG, TC, and LDL levels in negative control animals. On the administration of *C. afer* extract, there was a significant improvement in the lipid profile as indicated by lowering serum TAG, TC, and LDL levels to near normal [39]. Results from these studies indicate that *C. afer* plant leaves could be explored in the management of diabetes mellitus and its complications such as dyslipidemia.

#### 3.4.2. Protective Ability against Kidney, Liver, Heart, Testicle, and Mitochondrial Damage

When the body is exposed to toxins or drugs, it becomes imperative for organs such as the liver and kidney to detoxify such substances. The liver is usually involved in the biotransformation of toxins to less toxic compounds through phase I and II reactions to enhance their elimination by kidneys. In a disease state of the kidney, its detoxifying capacity is impaired. Toxicity of the kidney results in elevated concentrations of sodium and potassium in the serum and enlarged kidney. In cyclosporin-a- (Csa-) induced nephrotoxicity animal model, there is generally a significant elevation of serum K^+^, Na^+^, BUN, and creatinine in negative control animals compared to the normal rats. A report published by Ezejiofor et al. indicates that oral administration of aqueous *C. afer* leaf extract showed a significant dose-dependent reduction of serum BUN and K^+^ [40].

In a gentamicin-induced nephrotoxicity model involving oral administration of aqueous *C. afer* leaf extract, there was a significant decrease in sodium, blood urea level, and serum creatinine level. There was as well a significant decrease in serum potassium concentration, which occurred in a dose-dependent manner. The low and medium dose of aqueous leaf extract of *C. afer* reversed the deleterious effect of gentamicin on the kidney [41]. The *C. afer*-treated rats regained their normal kidney morphology with glomeruli and compact tissue appearance while the untreated rat showed the presence of dissolved cells due to necrosis of the nephron and widespread infiltration of inflammatory cells. There was an observed improvement in tissue architecture with visible glomeruli and less cell inflammation when *C. afer* leaf extract with doses of 375–1125 mg/kg was administered [41]. This depicts the fact that *C. afer* leaves have nephroprotective property.

Another study reported by Ezejiofor and Orisakwe in 2019 also noticed the ability of *C. afer* in lead-induced kidney damage model. The study observed a significant reversal in the decreased levels of glutathione peroxidase (GSH-PX), superoxide dismutase (SOD), catalase (CAT), glutathione S-transferase activity (GST) seen in the lead acetate only treated group [42]. This observation shows that *C. afer* seems to have a nephroprotective effect against lead-induced damage in a male albino rat model.

The role of the liver is so critical that there is a need to protect it from damage. One of the causes of damage to the liver is oxidative stress and its close proximity to the intestines makes it more prone to a wide spectrum of food-borne toxins. The damaging effect of free radicals to the liver is increased during exposure to xenobiotic such as drugs. A defect in the liver leads to the leakage of certain liver enzymes into the blood. These enzymes include alanine aminotransferase, alkaline phosphatase, and aspartate aminotransferase. These enzymes therefore serve as biomarkers for the detection of liver damage in serum. In a study model involving induced liver injury by the oral administration of carbon tetrachloride (CCl_4_) in rats, there was a significant reduction in serum AST and alkaline phosphatase (ALP) close to the normal at an oral administration of 400 mg/kg of *C. afer* extract compared to rat fed with 200 mg/kg of the extract [8]. In another study, it was demonstrated that *C. afer* stem extract possesses the ability to protect against alcohol-induced liver damage in rats [43], indicating that *C. afer* has pharmacological activity against alcohol liver cirrhosis.

In a cardiac-induced toxicity with CCl_4_ (2.5 mL/kg) with olive oil in a ratio of 1 : 1, there is usually formation of CCl_3_OO radical that causes the peroxidation of polyunsaturated fatty acids associated with the cardiac cell. In a study involving CCl_4_-induced cardiac toxicity, administration of *C. afer* leaf extract proved protective [38]. This was depicted by a significant improvement in the lipid profile as indicated by low serum TAG, TC, and LDL levels to near normal.

Lead (Pb) is a multiple organ toxicant and an oxidative stress inducer. The protective effect of aqueous leaf extract of *C. afer* on Pb-induced testicular damage was evaluated in a study reported by Ezejiofor and Orisakwe in 2019 [44]. The result showed insignificant changes in the weights of epididymis and testes when extract treated Pb group was compared with the normal control. Marked rise was noted in the sperm analysis, blood Pb level, and luteinizing hormone (LH) and a decrease was observed in follicle-stimulating hormone (FSH) with nonsignificant changes in testosterone in the extract treated Pb group compared to the normal control. The outcome according to the researchers depicts the fact that aqueous leaf extract of *C. afer* may be protective against lead-induced testicular damage.

Cadmium toxicity is generally treated with chelating agents such as dimercaprol mesomercaptosuccinic acid and ethylenediaminetetraacetic acid (EDTA) which are commercially available, and results of many histological studies have shown that the chelating agents are not safe. *C. afer*, a plant natural agent, was explored in a study to ascertain its potential in protecting against Cd-induced reproductive toxicity in male rats. The results reported in by Atere and Akinloye [45] are at variance with what Ezejiofor and Orisakwe also reported when they used lead as the toxic agent [44]. The antioxidant role of *C. afer* plant extract in these pharmacological activities cannot be overemphasized as there are several published reports to substantiate it [2, 7, 9, 25, 40, 43].

Another report by Anyasor and colleagues published in 2017 [46] shows a chemoprotective activity of *C. afer* against Ca^2+^-induced mitochondrial membrane permeability transition (MPT). According to the report, both *C. afer* leaf and stem extracts obtained from different solvents notably inhibited Ca^2+^-induced mitochondrial MPT when compared with an untreated mitochondrial fraction in the presence of Ca^2+^ ion [4, 46]. In a study, *C. afer* hexane leaf extract at a dose of 10–60 *μ*g/ml significantly inhibited Ca^2+^-induced MPT by 85.66% to 90.45% [46]. This suggests that *C. afer* contain certain bioactive compounds that chelate calcium ion to help stabilize the membrane, thus inhibiting mitochondrial MPT. This indicates that *C. afer* leaves could help cure and manage oxidative stress and neurodegenerative diseases such as Alzheimer's disease.

Almost all the pharmacological effects observed in the chemical-induced organ damage models have been attributed to antioxidant or free radical scavenging potential as demonstrated by Tchamgoue and colleagues [47]. Many studies have demonstrated the *in vitro* and *in vivo* antioxidant abilities of *C. afer*. In an *in vivo* study on cardiac toxicity, for example, there was a significant reduction in lipid peroxidation, improvement in lipid profile, and increase in antioxidant activity [38]. There was also a decrease in the creatine kinase (CK) and aspartate aminotransferase (AST) levels. Meanwhile, there was an improvement in the activity of superoxide dismutase (SOD), catalase (CAT), and glutathione S-reductase (GSR) which shows that *C. afer* extract renders cardiac protection against toxicity [38]. In an *in vivo* nephrotoxicity model, *C. afer* aqueous leaf extract significantly resulted in an increase in GSH, CAT, SOD, and GST (glutathione S-transferase) activity in rats [40].

*In vitro* antioxidant studies on aqueous stem extract of *C. afer* showed an IC_50_ value of 92.33 mg/mL for DPPH (diphenylpicrylhydrazyl) radical [22]. The methanol extract of the *C. afer* leaf showed a DPPH scavenging activity with an IC_50_ of 157.48 *μ*g/mL [2]. Hexane leaf extract of *C. afer* is reported to have NO (nitric oxide) and H_2_O_2_ (hydrogen peroxide) scavenging activity with an IC_50_ of 1.77 mg/mL and 0.33 mg/mL, respectively [7, 9, 25]. All these data depict the fact that *C. afer* plant could be considered in the drug discovery pipeline as a candidate for protective agent against liver, kidney, heart, testicles, and mitochondrial damage.

#### 3.4.3. Analgesic and Anti-Inflammatory Effects

Analgesic is used as a painkiller without blocking any nerve impulse conduction, altering sensory perception nor affecting consciousness. Some of the methods employed in evaluating analgesic property include acetic acid-induced writhing, the tail flick assay, and the tail immersion assay. A study by Ijioma et al. [48] investigated the antinociceptive property of *C. afer* ethanol leaf extract and the stem juice in rats, using different rat models—acetic acid, tail flick, and tail immersion assays. The results indicate that both extract and stem juice of *C. afer* reduced the number of writhes and increased reaction time in the experimental rats exposed to pain stimuli. Another study [49] involving hexane, ethyl acetate, *n*-butanol, and aqueous fraction, the hexane fraction of *C. afer* leaf exhibited the highest antidenaturation of protein, stabilization of HRBC membrane, and antiproteinase activities when compared with other test fractions. The study further identified compounds, which include phytol, coumaran, hexadecanoic acid, octadecatrienoic acid, and *cis*-vaccenic acid as some key compounds responsible for the anti-inflammatory effects of the plant.

Momoh and colleagues in 2011 [50] had reported that *C. afer* contain phytochemical substances including alkaloids, flavonoids, tannins, phenols, glycosides, and terpenoids, some of which have been implicated in antinociception. According to Ijioma et al., the possible mechanisms of action of the leaf and stem juice of *C. afer* include inhibition of the activity of cyclooxygenase-2 (cox-2) and interference with G-protein-mediated signal transduction.

Inflammation can be defined as the body's attempt at self-protection with the aim of removing harmful stimuli including damaged cells, irritants, or pathogens and begins the healing process. It is one of the first lines of defense the body adapts to localize and destroy invaded microorganisms or neutralize chemical toxins. Earlier reports by Moody and Okwagbe [51] had hinted that the chloroform and methanol extracts from the aerial parts displayed a noteworthy reduction in carrageenan-induced rat paw edema, while the methanol extract of the rhizome exhibited marked topical anti-inflammatory effect in croton aldehyde-induced mouse ear edema.

In another study involving arthritis-induced rat model, data collected revealed a significant reduction in plasma total bilirubin, total protein, and globulin concentration in rats treated with *C. afer* hexane leaf fraction compared with negative control [9]. On oral administration of the plant extract, there was a significant reduction of carrageenan-induced rat paw edema as compared with the negative control group. The inhibition of the paw edema was concentration-dependent [9]. In the arthritis-induced rat model, the oral administration of *C. afer* hexane leaf fraction showed a significant reduction in white blood cells (WBCs), lymphocytes, neutrophils, basophils, eosinophils, monocytes, and blood platelet count compared with the elevated form in the negative control group. This depicts the fact that *C. afer* leaf possesses immunomodulation activity against proinflammatory mediators. The hexane fraction of the plant is reported to have high antidenaturation effects on protein, stabilization of HRBS membrane, and antiproteinase with IC_50_ values of 33.36 *μ*g/mL, 33 *μ*g/mL, and 212.77 *μ*g/mL, respectively [9]. Data reported by Anyasor and colleagues in 2014 [9] linked the anti-inflammatory effects to a boost in the antioxidant status *in vivo*. The *in vivo* antioxidant study showed that *C. afer* hexane extract-treated animals had a marked elevation in the levels of GSH, SOD, CAT, and GST activities while the level of MDA was significantly reduced in the plasma, liver, kidney, and brain.

Reports seen in the analgesic and anti-inflammatory experiments seem to provide some evidence to support the traditional use of *C. afer* extract for the relief of pain and inflammations in the management of arthritis. Implicitly, the plant could be considered as a drug candidate in pain and inflammatory drug development.

#### 3.4.4. CNS Depressant Activity

The use of central nervous depressants such as phenobarbitone decreases the transmission of impulse between the receptor organs and the effector organs mediated by the brain. In mechanism, the phenobarbitone inhibits GABA receptors and further inhibits chloride current through the receptor channels. That is, the glutamate-induced depolarization is inhibited by the phenobarbitone, thereby causing hypnosis [52]. According to Ezejiofor and Igweze, 300 mg/kg has a little effect on the gait at 90 and 120 minutes compared with the control. There was also a slight effect of 800 mg/kg of *C. afer* extract on the gait at 60, 90, and 120 minutes and on its movement at 120 minutes. However, there was a potentiating effect of the plant extract on the standard drug in a concentration-dependent manner. The combination of phenobarbitone and extract (300 and 800 mg/kg) showed a significant CNS activity [52]. The results reported hints of a need for more research on CNS depressant effects of *C. afer*.

#### 3.4.5. Antiparasitic and Antibacterial Property

*Aedes aegypti* is a mosquito, which is known to spread yellow fever, dengue fever, chikungunya, and zika fever. These mosquitoes are commonly noticed in the tropical and subtropical region including Africa. The conventional agents against these mosquitoes are limited and pose adverse effects on both the individual and the environment. One of the routes to reduce the spread of these diseases by these mosquitoes is through drug agents, which have larvicidal activity against larvae of *Aedes aegypti*. The eggs of *A. aegypti* were suspended in water and fed with rabbit pellet until the larvae grew to the 4^th^ instar stage. The leaf and stem extract at a concentration range of 0–5 mg/mL was used to assess the larvicidal activity. It was found that the LC_50_ values (in both leaf and stem extract) were relatively higher in those exposed to the extract for 28 hours than for 48 hours. The same trend was observed for the reported LC_90_. This indicates that the long exposure (administration of *C. afer* extract) augments its larvicidal activity. The LC_50_ was found to be 8.24 ± 1.15 and 9.00 ± 0.2 at 24 hr and 4.06 ± 0.27 and 3.79 ± 0.05 at 48 hr [53], respectively.

According to Moundipa and colleagues [53], *C. afer* has a mortality effect against *Entamoeba histolytica* which showed an EC_50_ of 11.43, 11.02, and 191.10 *μ*g/mL for two, four, and six hours, respectively.

The antibacterial activity of the ethanol extract of *C. afer* has also been reported. The data available indicate that extract possesses moderate inhibitory activity against *Staphylococcus aureus* compared to the reference drug [29]. According to Ezejiofor et al., leaf extract showed lower inhibition of the Gram-negative organisms (*Pseudomonas aeruginosa* and *Escherichia coli*) compared to the reference drug [54]. The data reported in the literature hints of a need for more research on antibacterial properties of *C. afer* with regard to other pathogenic bacteria.

### 3.5. Toxicological Effects of *Costus afer*

Exogenous substances such as xenobiotic usually pose toxicity to some vital organs in the body even though these organs try to get rid of the toxins. Many drugs are taken to cure and manage diseases and disorders and are seen to be effective at its purpose, but one cannot overlook its side effects. In an acute toxicity study with *C. afer* hexane leaf extract, male rats were orally given 2000 mg/kg of *C. afer* hexane leaf fraction after overnight fasting. After 24 hours, the rats were observed for clinical signs in fur color, breathing rate, and death. There were however no clinical signs of toxicity nor death, indicating that *C. afer* leaf extract is safe even at a dosage of 2000 mg/kg bwt [12, 55, 56].

In a subchronic toxicity study, there was a significant reduction of hemoglobin (HB) and total red blood cell (RBC) count, indicating that long-term use of aqueous leaf extract of *C. afer* could result in anemia. Further studies need to be done to investigate the bioactive compounds in the plant responsible for the decreased in Hb and total RBC count as well as the type of anemia caused [12]. It was also revealed that aqueous leaf extract of the plant triggered an increase in weight of the liver and other clinical chemistry parameters such as ALT, ALP, AST, and TB but not the kidney. The histological study also showed architectural and vacuolar changes in the hepatic cells, which was concentration-dependent. This was more prominent at a dose of 1125 mg/kg. There was no observed detrimental histological effect/defect on the kidney [55].

In an abortifacient study reported by Anaga et al. [19] where virgin female rats administered with *C. afer* leaf extract were allowed to mate with the male rat, expulsion of the fetuses wrapped in the placenta in the 3^rd^ trimester of pregnancy was observed and reported to be 100% in the studied rats.

## 4. Concluding Remarks

The pharmacological property of the leaf, stem, and rhizome of *C. afer* compiled in this piece provides some scientific evidence for its use as an antihyperglycemic, hepatoprotective, cardioprotection, nephroprotective, analgesic, anti-inflammation and antioxidant, and other pharmacological activities. All these findings support its use in the management of various diseases, thus demonstrating its wide-ranging clinical relevance. The compilation of information on *C. afer* plant parts used for the management of many diseases can serve to promote a more rational medicinal use of the plant. It can also offer evidence-based data for clinical development of the medicinal plant. The presence of high proportions of micro- and macronutrients such as carbohydrates and proteins makes it possible for the consideration of the plant as a nutraceutical. This and the evidence of abundance of phytochemicals are suggestive of the reasons why local folks use this plant. However, pregnant women must be cautioned about the use of the plant since it has the tendency to induce abortion in the 3^rd^ trimester. The current search has exposed the inadequacy of information on the pharmacokinetics and pharmacodynamics of *C. afer* extracts and its isolated compounds; thus, a call for more research in that regard is needed.

## Figures and Tables

**Table 1:** Names of *Costus afer* in the dialects of some selected West African countries.

Country	Tribe	Name of the plant	Reference
*Nigeria*	Yoruba	Irekeomede, Tete-egun	[1]
	Ijaw	Ogbodou	[6]
	Igbo	Opete or okpete, Ejula	[1, 12]
	Hausa	Kakizawa, Dodon kodi	
	Efik	Mbtitem	[4]

*Ghana*	Fante	Osommbaa	[13]
	Asante	Sommε	[14]
	Ewe	Asumbae	
	Nzema	Enyane	

*Cameroon*	Douala	Mwandando	[15]
	Bakweri	Mandanwany	[16]
	Bulu	Nmian	[17]

*Senegal*	Fogny	Bumay	
	Dan	Sungho	[14]
	Guere	Zazaboto	
	Kru-bete	Doï	

*Ivory Coast*	Guere do	Dodré	[14]
	Guere	Zazaboto	
	Maho	Yaya	
	Bulom	Sayina-lε	
	Gola	Sawa	

*Sierra Leone*	Kissi	Siaŋdẽ	[14]
	Kono	Tofa	
	Loko	Dan	
	Dan	Sungho	
	Tem	Bomire	

*Togo*	Edo	Úkhúerúohā	[14]

*Guinea-Bissau*	Fula-pulaar	Gògódje-súto	[14]

**Table 2:** Ethnomedicinal uses of *Costus afer*.

Country	Parts used	Reference
Inflammation	Stem	[5, 12]
Arthritis	Leave, stem	[3, 5, 18]
Stomach ache	Leaves	[18]
Cough, sore throat	Stem, aerial parts	[3, 12]
Measles	Leaves	[5]
Malaria	Rhizome	[18]
Chicken pox	Stem	[17]
Influenza	Stem	[17]
Genital herpes	Stem	[17]
Fodder	Leaves, stem	[5]
Purgative	Rhizome	[5]
Laxative	Rhizome	[5]
Diabetes mellitus	Stem, leaves, rhizome	[12]
Wound healing	Leaves	[18]
Diuretic	Stem	[5, 12]
Aperient	Stem	[19]
Jaundice	Leaves	[18]
Fever	Leaves	[18]
Leprosy	Rhizome	[5]
Gastric ulcer	Rhizome	[20]
Colic	Leaves	[18]
Hypertension	Leaves	[5, 18]
Hemorrhoids	Stem	[5]
Toothache	Rhizome	[5]
CNS depression	Rhizome	[21]
Helminthic	Leaves	[9]
Hepatic disorder	Leaves	[9]
Miscarriage	Leaves	[9]
Gonorrhea	Stem	[16]
Aphrodisiac	Leaves, stem, rhizome	[22]
Ear infection	Leafy stem	[23]
Conjunctivitis	Leafy stem	[15]
Oligospermia	Leaves	[3]
